# TGF-β inhibitors: the future for prevention and treatment of liver fibrosis?

**DOI:** 10.3389/fimmu.2025.1583616

**Published:** 2025-06-27

**Authors:** Weili Wang, Yilin Gao, Yizhen Chen, Meng Cheng, Yonghao Sang, Liuting Wei, Rong Dai, Yiping Wang, Lei Zhang

**Affiliations:** ^1^ First Clinical Medical College, Anhui University of Chinese Medicine, Hefei, China; ^2^ Department of Nephrology, The First Affiliated Hospital of Anhui University of Chinese Medicine, Hefei, China

**Keywords:** liver fibrosis, TGF-β signaling pathway, TGF-β inhibitors, hepatic stellate cells, antifibrotic therapy

## Abstract

Liver fibrosis is a core pathological process in the progression of chronic liver diseases to cirrhosis and hepatocellular carcinoma, characterized by abnormal deposition of extracellular matrix. Transforming growth factor-β (TGF-β), through classical small mothers against decapentaplegic (Smad)-dependent and non-Smad-dependent pathways, activates hepatic stellate cells to transdifferentiate into myofibroblasts, promotes extracellular matrix synthesis, and regulates immunity, serving as a key driver of fibrogenesis. This review systematically summarizes the role of TGF-β in liver fibrosis and details the research progress of TGF-β-targeted inhibitors. Studies show that TGF-β neutralizing antibodies, small molecule receptor antagonists, small molecule signaling inhibitors, and natural compounds and extracts significantly improve experimental liver fibrosis by inhibiting Smad or non-Smad pathways. In clinical trials, drugs such as Pirfenidone and Hydronidone have demonstrated potential for fibrosis reversal in patients with chronic hepatitis. Although TGF-β-targeted therapy has made breakthroughs in basic research and clinical translation, future studies need to focus on multi-target drug design, personalized treatment regimens, and novel delivery systems to accelerate the transition from preclinical research to clinical application, providing innovative therapeutic strategies for liver fibrosis and related liver diseases.

## 1 Introduction

Liver fibrosis is a pathological process involving structural changes in liver tissue and excessive extracellular matrix (ECM) deposition due to chronic injury and inflammation (1). Liver fibrosis is a common complication of various chronic liver diseases (CLDs), such as viral hepatitis, fatty liver disease, and alcoholic liver disease, and represents the early stage of liver cirrhosis (2, 3). The progression of liver fibrosis typically impairs liver function and may further lead to liver cirrhosis, failure, and cancer (4). Data show that from 1999 to 2016, the number of deaths due to liver cirrhosis in the United States increased by 65%, reaching 34,174. The number of deaths from hepatocellular carcinoma (HCC) more than doubled, reaching 11,073 (5). In 2017, approximately 1.5 billion people worldwide were affected by CLD; thus, it is a substantial global public health issue (6). Liver fibrosis is a critical stage in liver disease progression, and current treatments are focused primarily on managing underlying diseases; effective drugs that directly target fibrosis are scarce. Transforming growth factor-beta (TGF-β) is a representative member of the TGF-β family, which also includes activins, nodal factors, bone morphogenetic proteins (BMPs), growth and differentiation factors (GDFs), and other related factors (7). TGF-β is widely recognized as a key mediator of tissue fibrosis, primarily through the activation of its downstream Smad signaling pathway, triggering the overexpression of profibrotic genes and further inducing scar tissue formation (8). Numerous studies have demonstrated that dysregulation of the TGF-β pathway is a critical pathogenic mechanism in tissue fibrosis (9–12), playing a central role in its initiation and progression (13). TGF-β activates hepatic stellate cells (HSCs), causing their transdifferentiation into myofibroblasts (MFBs) and leading to the excessive accumulation of ECM components such as collagen in the liver, thereby driving the progression of liver fibrosis (14, 15). Additionally, the TGF-β signaling pathway is involved in liver repair, immune responses, and cell apoptosis (16). Given the critical role of TGF-β in liver fibrosis, the use of TGF-β inhibitors has become an important research focus in treating liver fibrosis. In recent years, numerous studies have validated the potential of TGF-β-targeted inhibitors in treating fibrotic diseases (17–22), indicating that TGF-β inhibitors are widely regarded as promising antifibrotic therapies. With a deeper understanding of the mechanisms of the TGF-β signaling pathway, researchers have developed various TGF-β-targeted inhibitors and achieved preliminary results in different animal experiments and clinical trials. This review focuses on the biological processes of TGF-β, its dual role in the development of liver fibrosis, and the application of TGF-β inhibitors in liver fibrosis treatment, exploring previous research progress, clinical achievements, and future development trends.

## 2 Relationship between the TGF-β signaling pathway and liver fibrosis

### 2.1 TGF-β family

In mammals, the 33 genes of the TGF-β family each encode a polypeptide comprising a secretory signal peptide, a pro-domain of 1–250 residues, and a growth factor domain of 1–110 residues (23, 24). The TGF-β superfamily includes TGF-β, BMPs, GDFs, activins, and nodal factors, with TGF-β being the prototype. The three primary TGF-β protein isoforms are TGF-β1, TGF-β2, and TGF-β3 (25). As key members of the TGF-β family, these isoforms are widely involved in critical physiological processes, such as embryonic development, cell differentiation, organ formation, and tissue repair (26–29). Additionally, TGF-β plays a significant role in immune regulation by inhibiting T-cell proliferation, promoting the generation of regulatory T cells, and modulating the differentiation and function of Th1/Th17 cells, thereby profoundly influencing immune tolerance, autoimmune diseases, and tumor immunity (30, 31). The expression of TGF-β is regulated by various factors, including proinflammatory cytokines, oxidative stress, Toll-like receptor (TLR) signaling, the ECM, and matrix metalloproteinases (MMPs) (32, 33). TGF-β, which plays important roles in numerous physiological and pathological processes, is primarily produced by macrophages and epithelial cells; platelets, T cells, fibroblasts, and mast cells can also secrete this cytokine (34, 35).

**Figure 1:**
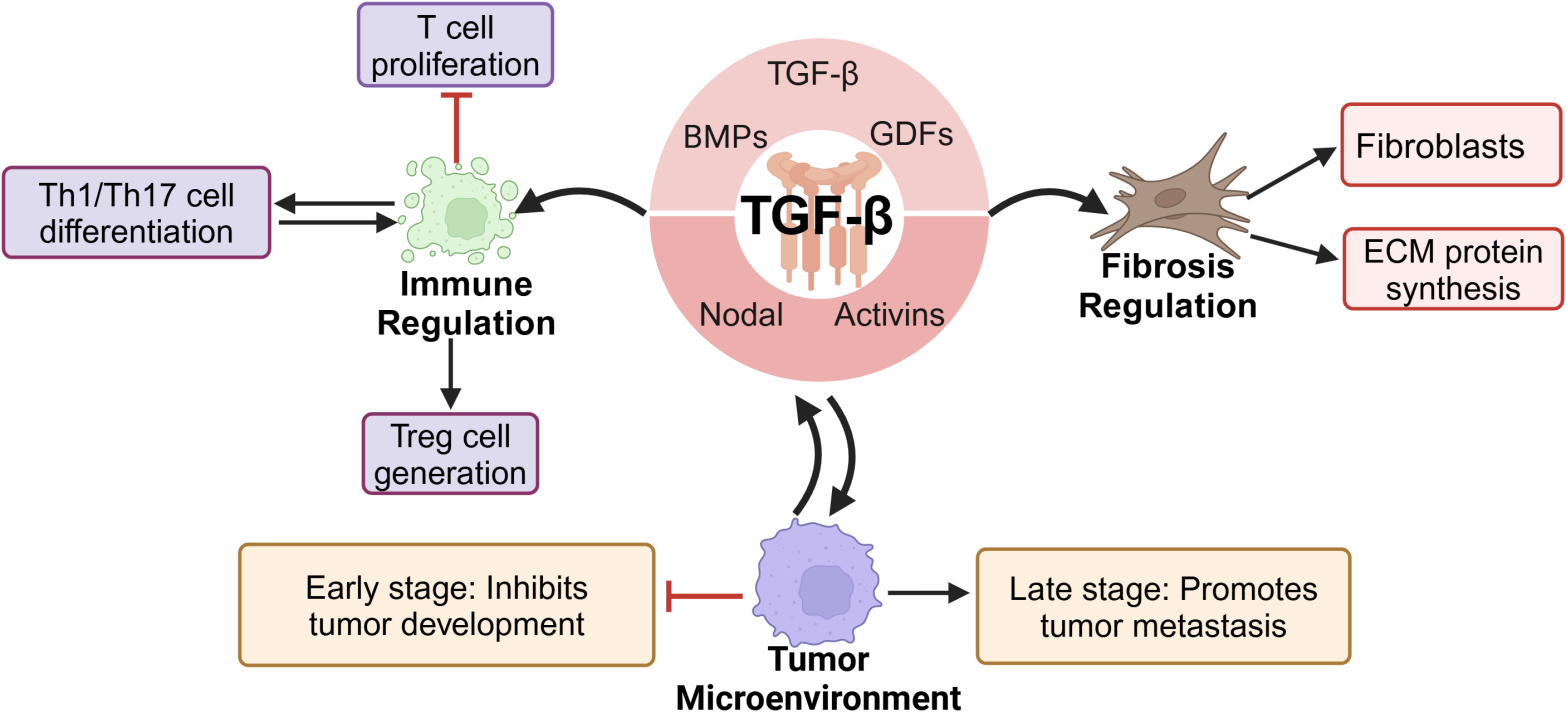
TGF-β family and its biological functions.

TGF-β is a key activator of fibroblasts and plays a central regulatory role in fibrotic responses. It directly promotes fibroblast activation and may further drive pathological progression by modulating the fibrotic phenotypes of immune cells and vascular cells (36, 37). Additionally, TGF-β stimulates the synthesis of ECM proteins and induces tissue fibrotic responses *in vivo* (38). In fibrotic diseases, TGF-β promotes the proliferation and activation of fibroblasts by inducing the transdifferentiation of HSCs, lung fibroblasts, and renal tubular epithelial cells into MFBs, leading to excessive collagen and ECM deposition, ultimately resulting in organ fibrosis. Therefore, TGF-β is considered a key pathogenic factor in fibrotic diseases of the liver, lungs, kidneys, and other organs (8, 39–41). Furthermore, TGF-β plays a dual role in the tumor microenvironment. In early tumorigenesis stages, TGF-β is suppressive; in the advanced stages, it promotes immune evasion, angiogenesis, and tumor metastasis, driving cancer progression (42, 43) (**Figure 1**).

### 2.2 TGF-β signaling pathway

TGF-β ligands initiate downstream signaling by binding to their receptors, TGF-β receptor type I (TGF-βR I) and type II (TGF-βR II), which are primarily involving two pathways. Among these pathways, the canonical small mothers against decapentaplegic (Smad)-dependent pathway is crucial to TGF-β signaling. Smads are key transducers of the TGF-β family signaling pathway and are divided into three main subclasses: receptor-regulated Smads (R-Smads), common Smads (Co-Smads), and inhibitory Smads (I-Smads) (44, 45). R-Smads include Smad2, Smad3, Smad1, Smad5, and Smad8, with Smad2 and Smad3 being the key downstream mediators of TGF-β-induced tissue fibrosis (46). In this pathway, the TGF-β-activated receptor complex phosphorylates R-Smads, which then form a complex with Co-Smad4. This R-Smad–Co-Smad complex translocates to the nucleus and regulates the transcription of target genes (47, 48). Smad1, Smad5, and Smad8, which are R-Smads, primarily participate in BMP signaling, regulating osteoblast differentiation and bone and cartilage development (49, 50). Within the I-Smad family, Smad6 and Smad7 function as negative regulators of the TGF-β/Smad signaling pathway. Specifically, Smad7 inhibits pathway activation by binding to activated TGF-β type I receptors, thereby blocking Smad2 phosphorylation. Additionally, its MH2 domain (Mad Homology 2 Domain) mediates specific binding to TGF-β/BMP receptor complexes, further suppressing signaling from the TGF-β superfamily (51–53).

**Figure 2:**
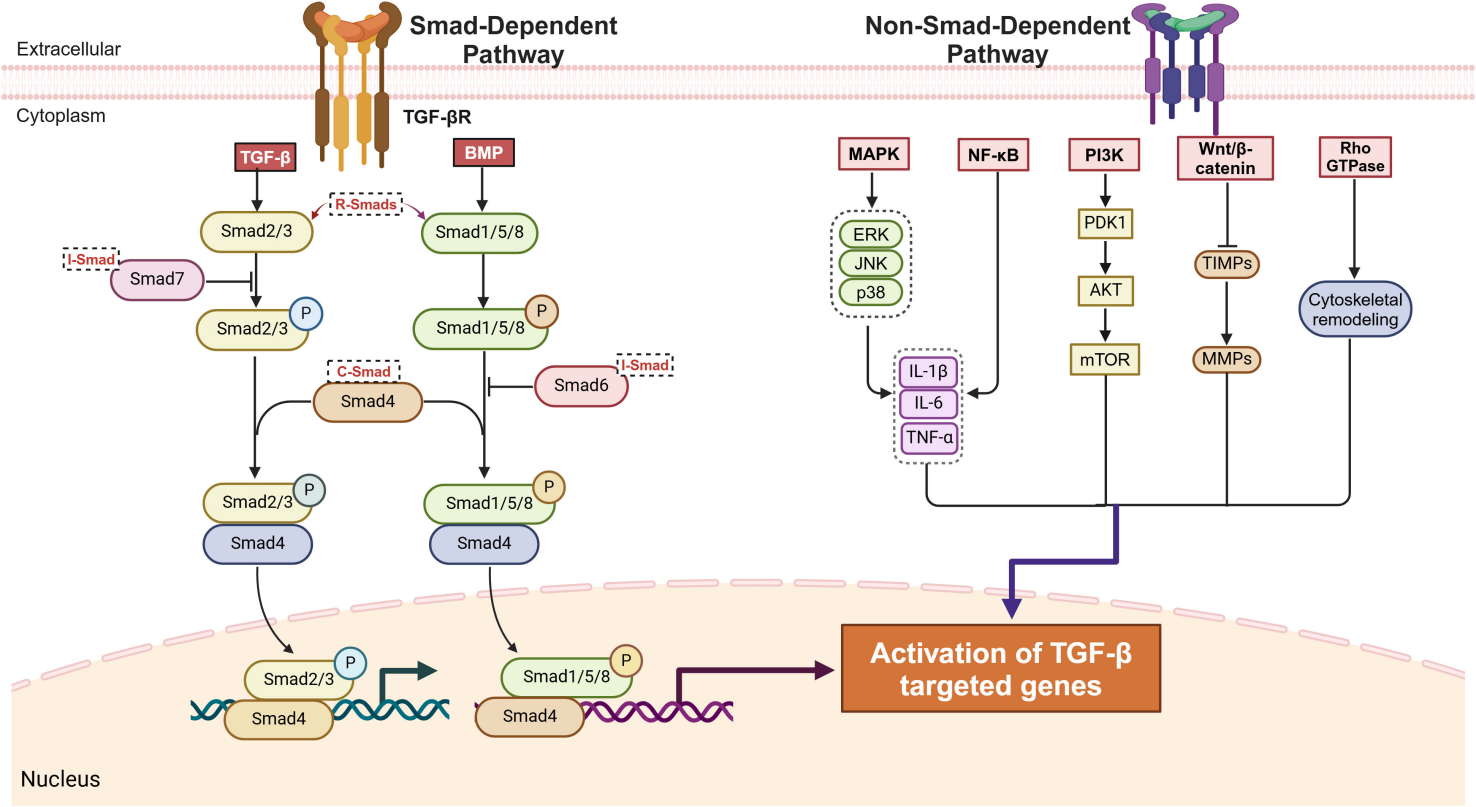
TGF-β signaling pathway: smad-dependent and non-smad-dependent mechanisms.

The other pathway is the non-Smad-dependent pathway, in which TGF-β activates signaling cascades through phosphorylation, acetylation, sumoylation, ubiquitination, and protein–protein interactions. These pathways include mitogen-activated protein kinase (MAPK), phosphoinositide 3-kinase/protein kinase B (PI3K/Akt), ras homolog family GTPase (Rho GTPase), nuclear factor kappa-light-chain-enhancer of activated B cells (NF-κB), wingless-related integration site/β-catenin (Wnt/β-catenin), mammalian target of rapamycin (mTOR) (54, 55). These non-Smad-dependent pathways interact with Smad signaling to jointly regulate fibroblast proliferation, fibrosis-related gene expression, and ECM remodeling, significantly driving fibrosis initiation, progression, and disease development (56, 57) (**Figure 2**).

### 2.3 Pathogenesis of liver fibrosis

Liver fibrosis represents a reversible wound-healing response characterized by abnormal deposition of ECM triggered by chronic liver injury, which may progress to cirrhosis and even liver cancer (58, 59). While mild and transient liver injury allows the liver to restore normal structure through robust self-healing capacity, persistent damage induces chronic inflammation and excessive ECM accumulation, leading to gradual replacement of normal hepatic parenchyma by fibrotic scar tissue and eventual progression to cirrhosis (60). Hepatic parenchyma is composed primarily of hepatocytes and liver sinusoidal endothelial cells, whereas non-parenchymal cells include HSCs, Kupffer cells, and infiltrating immune cells. The development and progression of liver fibrosis depend fundamentally on intercellular interactions among these cell types (61).

**Figure 3:**
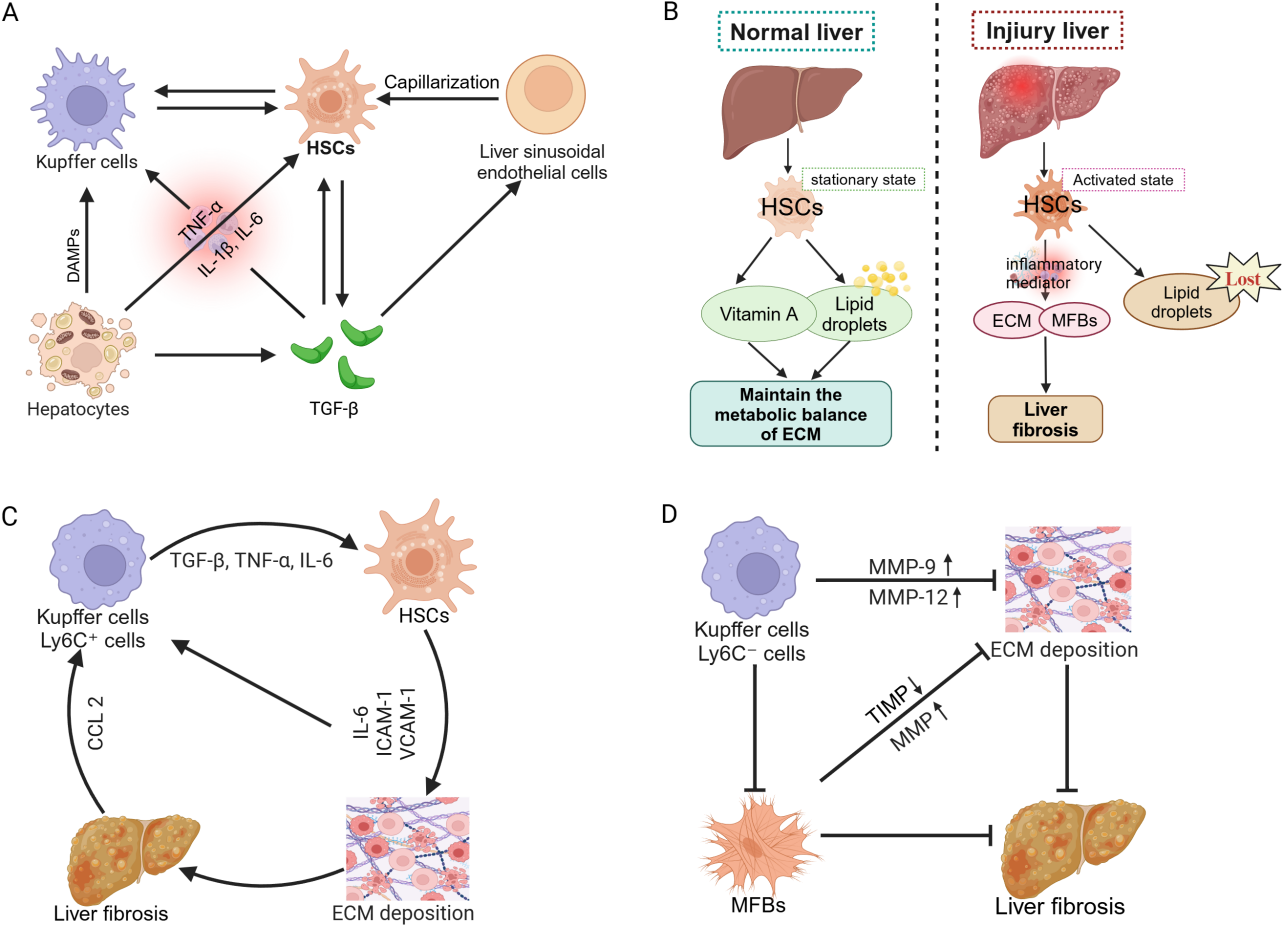
Pathogenesis of liver fibrosis. **(A)** Cellular interactions and progression mechanisms of liver fibrosis. **(B)** Role of HSCs in normal and injured liver. **(C)** Pro-inflammatory role of macrophages in liver fibrosis progression. **(D)** Macrophage-mediated reversal mechanism of liver fibrosis.

Kupffer cells, as resident macrophages located on the surface of hepatic sinusoidal endothelial cells (62), collaborate with infiltrating inflammatory cells during liver injury to release proinflammatory cytokines that activate HSCs and induce their transdifferentiation into MFBs (63). Activated HSCs lose their original stellate morphology and intracellular vitamin A-laden lipid droplets, instead vigorously synthesizing ECM components and secreting proinflammatory mediators, thereby forming the core driver of fibrogenesis (64). Concurrently, capillarization of liver sinusoidal endothelial cells—characterized by basement membrane thickening and loss of fenestrated structures—diminishes their normal capacity to regulate HSCs quiescence (65), further exacerbating ECM deposition and fibrotic progression (**Figure 3A**). The stark contrast between the quiescent phenotype of HSCs (vitamin A storage, lipid droplet-rich) and their activated state (ECM production, proinflammatory signaling) during this process (**Figure 3B**) constitutes the central pathological mechanism underlying the initiation and development of liver fibrosis.

During fibrosis progression, large numbers of Kupffer cells and Ly6C^+^ proinflammatory monocyte-derived macrophages are recruited to the liver via C-C motif chemokine ligand 2 (CCL2)-mediated chemotaxis. These cells release proinflammatory factors such as TGF-β and tumor necrosis factor-alpha (TNF-α), further activating HSCs and accelerating ECM accumulation. Additionally, HSCs secrete interleukin-6 (IL-6), intercellular adhesion molecule-1 (ICAM-1), and vascular cell adhesion molecule-1 (VCAM-1), enhancing interactions with macrophages and T cells (66), thus amplifying profibrotic signaling and promoting disease progression (**Figure 3C**). However, during fibrosis regression, Kupffer cells and monocyte-derived macrophages transition into Ly6C^−^ anti-inflammatory macrophages, releasing MMP-9 and MMP-12 and thus promoting ECM degradation and liver tissue repair (67). Simultaneously, the reduction in MFBs and TIMP levels further enhances MMP activity, accelerating ECM clearance and ultimately facilitating fibrosis reversal (68) (**Figure 3D**). In conclusion, macrophages play dual roles in the progression and regression of liver fibrosis.

### 2.4 Role of TGF-β in liver fibrosis

As a key regulatory factor in liver fibrosis, TGF-β drives the fibrogenic process through the TGF-β/Smad signaling pathway, which regulates DNA synthesis to promote the transformation of quiescent HSCs into MFBs. This process accelerates the excessive deposition of ECM and propels fibrosis progression. Concurrently, activated HSCs upregulate TGF-β1 expression via autocrine and paracrine mechanisms, forming a positive feedback loop that sustains the activation of the TGF-β signaling cascade (69). This process is mediated by the binding and activation of TβRI, which subsequently induces the phosphorylation of Smad2 and Smad3, leading to the formation of the Smad2/3/4 complex, which translocates to the nucleus. This complex triggers the transcription of fibrotic genes, including type I and type III collagens, ultimately resulting in ECM accumulation (39). However, Smad7, an endogenous inhibitor of the TGF-β signaling pathway, can competitively bind to TβRI, blocking TGF-β-mediated signaling and suppressing HSCs activation, thereby limiting fibrosis progression to some extent (70, 71). TGF-β also interacts with other signaling pathways, including the Notch, Wnt/β-catenin, and yes-associated protein/transcriptional co-activator with PDZ-binding motif (YAP/TAZ) pathways, further enhancing the profibrotic capacity of HSCs and exacerbating hepatocyte apoptosis and tissue damage (72–74).

**Figure 4:**
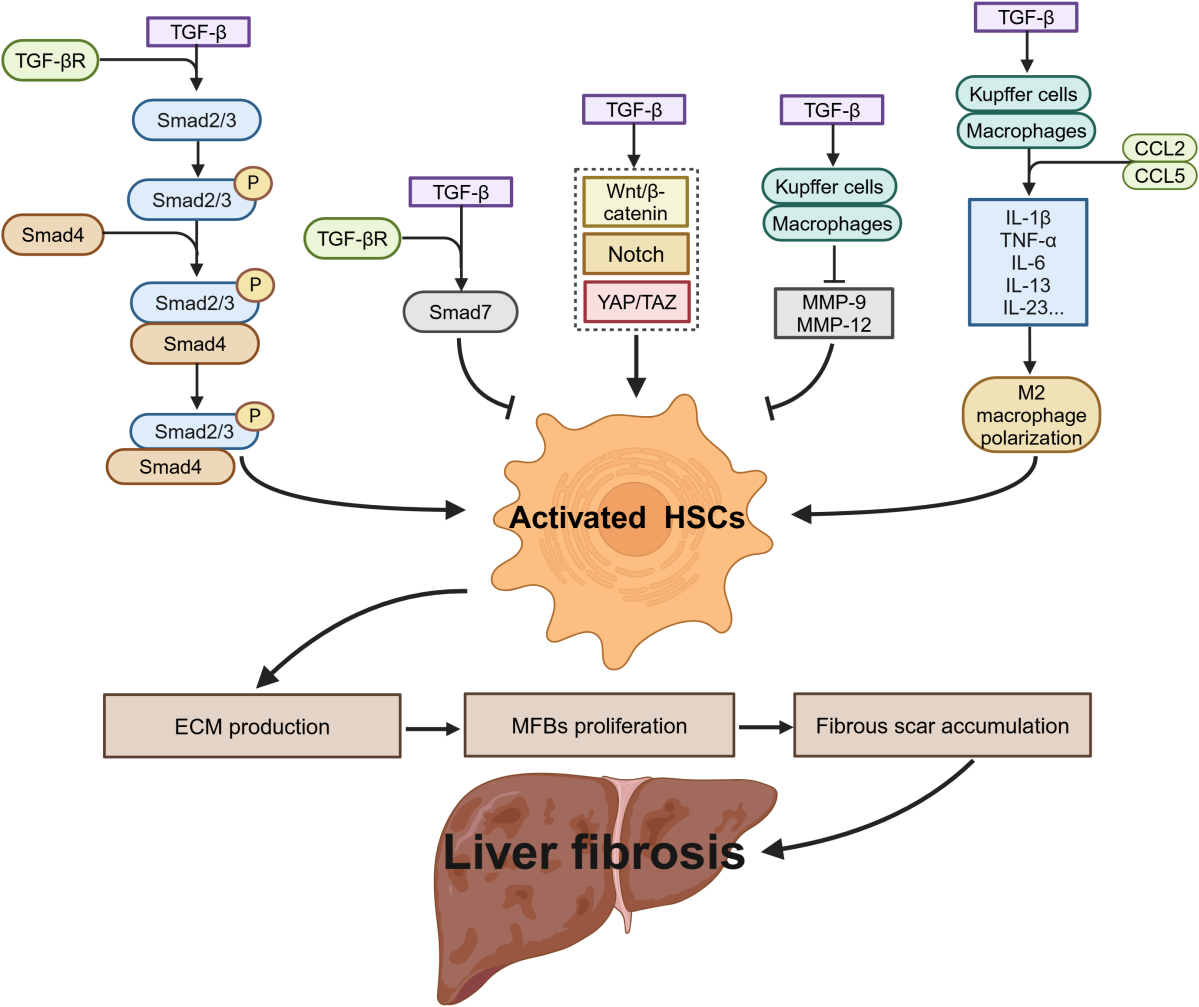
The role of TGF-β in liver fibrosis.

In the context of immunoregulation, TGF-β regulates the progression of liver fibrosis in multiple stages. In the early stages of fibrosis, TGF-β promotes Kupffer cell and macrophage secretion of proinflammatory cytokines, such as IL-1β, TNF-α, and IL-6, and the chemokines CCL2 and CCL5. These factors activate paracrine protective or apoptotic signaling pathways in hepatocytes and recruit additional immune cells, thereby aggravating inflammation and exacerbating liver damage (75). As fibrosis progresses to the later stages, TGF-β induces M2 macrophage polarization, enhancing immune suppression and facilitating the occurrence of HCC (76). In the fibrosis resolution phase, TGF-β may promote the production of MMP-9 and MMP-12, accelerating ECM degradation and facilitating fibrosis regression (77, 78) (**Figure 4**). Given TGF-β’s involvement in key processes of liver fibrosis, including HSCs activation, ECM production, immunoregulation, and fibrosis reversal, TGF-β and its associated signaling pathways have emerged as critical therapeutic targets. Various interventions have been developed, such as TGF-β-neutralizing antibodies, small molecule TGF-β receptor antagonists, small molecule TGF-β signaling inhibitors, and natural compounds, all aiming to block its profibrotic effects, promote reversal of liver fibrosis, and reduce the risk of liver cancer.

## 3 Research progress of TGF-β inhibitors in liver fibrosis

### 3.1 TGF-β inhibitors: types and molecular mechanism

#### 3.1.1 TGF-β neutralizing antibodies

TGF-β neutralizing antibodies directly bind to TGF-β, blocking its interaction with receptors and inhibiting fibrosis progression (**Table 1**). For example, BI 113823, a B1R small-molecule nonpeptide orally active inhibitor, can inhibit TGF-β-induced activation, proliferation, migration, and fibrotic protein expression in human hepatic stellate cells (hHSCs) and suppress the activation of the PI3K/Akt signaling pathway (79). Additionally, peptide seq-1 targets HepG2 human hepatocellular carcinoma cells (HepG2 cells) to negatively regulate the TGF-β signaling pathway, reducing the synthesis of ECM and alleviating the imbalance of ECM-remodeling-related enzymes MMP-2 and TIMP-2. This effect subsequently inhibits the activation, oxidative stress, and fibrogenic responses of HSCs via paracrine signaling mediated by conditioned medium from seq-1-treated HepG2 cells (80). The bifunctional fusion protein Bintrafusp alfa simultaneously targets TGF-β and programmed death-ligand 1 (PD-L1), significantly promoting T cell activation, regulating ECM deposition, and inhibiting fibrogenesis *in vitro*. Studies have shown that bintrafusp alfa is more effective than monotherapy, as it can neutralize both circulating free TGF-β and locally activated TGF-β on target cell surfaces (81, 82).

**Table 1:** Types and mechanism of action of TGF-β inhibitors.

Types of TGF-β inhibitors	Drug	Animal model types	Cell types	Mechanism	Molecular expression alterations	References
TGF-β neutralizing antibody	BI 113823	1.Carbon tetrachloride-induced mice model 2.Bile duct ligation-induced mice model	1.LX2 hHSC 2.Monocyte 3.Neutrophils	Inhibition of the PI3K/Akt signaling pathway	TGF-β↓, DBK↓, PDGF↓, CTGF↓, VEGF↓, B1Rs↓, P-Akt↓, α‐SMA↓, Col-1, 3, 4↓, IL-1↓, IL-6↓, MCP-1↓, MCP-3↓, TIMP-1↓, TNF-α↓	(79)
TGF-β neutralizing antibody	Peptide Seq-1	―	1.HepG2 2.LX2 hHSC	Inhibition of the TGF-β/Smand signaling pathway	TGF-β↓, ACTA2↓, Colla1↓, MMP-2↓, TIMP-2↓, ROS↓, IL-10↓, ALT↓, AST↓	(80)
TGF-β neutralizing antibody	Bintrafusp alfa	MC38 tumor-bearing mice	1. TIL 2. Detroit 562 cell	Inhibition of TGF-β and PD-L1	TGF-β↓, Colla3↓	(81, 82)
Small molecule TGF-β receptor antagonists	AZ12601011	Paraquat-induced mice model	―	Inactivation of TGF-β/Smad3	α-SMA↓, Col-1↓, IL-1β↓, IL-6↓, TNF-α↓	(83)
Small molecule TGF-β receptor antagonists	HTH-01-015	Carbon tetrachloride-induced mice model	LX2 hHSC	Inhibition of TGF-β/Smad2/3 signaling pathway and promotion of TGF-βR II/Smad4 degradation	α-SMA↓, Col-1↓, PAI-1↓, ALT↓, AST↓	(86)
Small molecule TGF-β receptor antagonists	N-butyl-fluoperazine iodide	1.Carbon tetrachloride-induced mice model 2.Thioacetamide-induced mice	1.LX2 hHSC 2.Primary mouse hepatic stellate cells	Inhibition of the TGF-β/Smad signaling pathway	TGF-βR II↓, P-Smad2/3↓, c-Jun↓, α-SMA↓, FN-1↓, PAI-1↓, ACTA2↓, Col1a1↓	(87)
Small molecule TGF-β receptor antagonists	J-1048	Thioacetamide-induced mice model	HSC	Inhibition of the TGF-β/Smad signaling pathway	TGF-βR I↓, P-Smad2/3↓, Smad7↑, α-SMA↓, Col-1↓, ALK5↓, P38α MAP↓, NLRP3↓, IL-1β↓	(88)
Small molecule TGF-β receptor antagonists	Galunisertib	Carbon tetrachloride-induced mice model	―	Targeting ALK5 to inhibit TGF-β/Smad2/3 signaling pathway	α-SMA↓, Col1a↓, FN-1↓, CTGF↓, MMP-1↑, Decorin↓	(91)
Small molecule TGF-β receptor antagonists	J-1155, J-1156	Thioacetamide-induced mice model	HSC-LX2	Inhibition of the TGF-β/Smad signaling pathway and blockade of the P2X7R-NLRP3 inflammasome axis	TGF-βR I↓, p-Smad2/3↓, Smad4↓, P2X7R↓, NLRP3↓, IL-1β↓, α-SMA↓, Col-1↓, Smad7↑, Smurf1/2↑	(92)
Small molecule TGF-β signaling inhibitors	12a and 12h	―	LX2 hHSC	Inhibition of the TGF-β/Smad signaling pathway	α-SMA↓, Col1a1↓, FN↓,	(93)
Small molecule TGF-β signaling inhibitors	Z-RIPΔ	Carbon tetrachloride-induced mice model	1.HSC-T6 2.LX2 hHSC	Inactivation of the TGF-β1/Smad and P38 MAPK signaling pathways	α-SMA↓, FN↓, P-Smad2/3↓, P-P38↓	(94)
Small molecule TGF-β signaling inhibitors	SIRT6 and SIRT7	1.Myeloid cell-specific knockout mice 2.Sirt6 whole-body knockout mice	LX2 hHSC	Inactivation of the TGF-β/Smad2/3 signaling pathway	α-SMA↓, P-Smad2/3↓, Col1a1↓, Col1a2↓, Col3a1↓	(95, 96)
Small molecule TGF-β signaling inhibitors	Arbidol	Bile duct ligation-induced mice model	―	Inactivation of the TGF-β/Smad2/3 signaling pathway	TGF-β1↓, P-Smad2/3↓, PDGFRII↓, PDGFRII↓, TGF-βr II↓, MMP-2↓, MMP-9↓, Col-1↓, ALT↓, AST↓	(97)
Small molecule TGF-β signaling inhibitors	ZINC62678696	1.Carbon tetrachloride-induced mice model. 2.Bile duct ligation-induced mice model	1.Murine immortalized macrophages RAW264.7 2.Human umbilical vein endothelial cells	Inhibition of the JNK, NF-κB and TGF-β signaling pathways	TGF-β1↓, TGF-β3↓, TNK/NF-kB↓, TNF-α↓, IL-6↓	(98)
Small molecule TGF-β signaling inhibitors	Empagliflozin	1.Mice model of non-alcoholic fatty liver 2.Methionine- and choline-deficient diet-induced fibrosis model	1.LX2 hHSC 2.Human hepatoma cell line	Inhibition of the TGF-β signaling pathway	miR-34a-5p↓, TGF-β↓, Grem2↓	(99)
Natural compounds and extracts	NR1	Carbon tetrachloride-induced mice model	1.Primary hepatic stellate cell 2.LX2 hHSC	Inhibition of the TGF-β1/Smad signaling pathway	PPAR-γ↑, TGF-βR I↓, P-Smad2/3↓, ALT↓, AST↓	(100)
Natural compounds and extracts	Curcumin	Carbon tetrachloride-induced mice model	LX2 hHSC	1.Activation of PI3K/Akt/mTOR signaling pathway 2.Inhibition of the TGF-β signaling pathway	H1↑, H2↑, KIF11↑, Dynein-3↑, TGF-β1↓, FN↓, HYP↓, Col-1↓, α-SMA↓, PDGFRB↓, TIMP-1↓, TLR-9↓, TGF-β↓	(101–104)
Natural compounds and extracts	Dihydrokaempferol	Carbon tetrachloride-induced mice model	1.LX2 hHSC 2.HepG2	Inhibition of the TGF-β1/Smad2/3, TNF-α and ERK1 signaling pathways	TGF-β1↓, P-Smad2/3↓, P-ERK1/2↓, α-SMA↓, Col-1/3↓, HYP↓, MDA↓, H2O2↓, SOD↑, IL-6↓, IL-1β↓, TNF-α↓, NF- kB↓, P65↓	(105)
Natural compounds and extracts	Raspberry unripe fruit extract	Carbon tetrachloride-induced mice model	―	Inhibition of the TGF-β/Smad signaling pathway	TGF-β1↓, P-Smad2/3↓, Smad4↓, HYP↓, α-SMA↓, Col1a1↓, TNF-α↓, MCP-1↓, IL-1β↓, IL-6↓, ALT↓, AST↓	(106)
Natural compounds and extracts	Sauchinone	Carbon tetrachloride-induced mice model	LX2 hHSC	Inhibition of the TGF-β/Smad/2/3 signaling pathway	α-SMA↓, PAI-1↓, MMP-2↓, P-Smad2/3↓, AVO↓, ROS↓, HIF-1α↓	(107)
Natural compounds and extracts	CPP-A-1	Carbon tetrachloride-induced mice model	LX2 hHSC	Inhibition of the TLR4/NF-kB and TGF-β1/Smad3 signaling pathways	TLR4/NF-kB↓, TGF-β1/Smad3↓, Col-1↓, α-SMA↓, MMP-1↓, TIMP-9↓, ALT↓, AST↓, TNF-α↓, IL-11↓, SOD↑, GSH↑, MDA↓	(108)
Natural compounds and extracts	LBPW	Carbon tetrachloride-induced mice model	LX2 hHSC	Inhibition of the TGF-β/Smad/2/3 signaling pathway	α-SMA↓, FN1↓, Col1a1↓, TGF-β1↓, TGF-βR II↓, P-Smad2/3↓, Smad7↑, MUC2↑, Occludin↑	(109)
Natural compounds and extracts	Esculetin	High fat diet-induced rat model	―	Inhibition of the PI3K/FoxO1, TGF-β and NF-kB signaling pathways	TGF-β↓, NF-kB↓, MMP-1↓, P-MEK1↓, P-ERK1↓	(110)
Natural compounds and extracts	Breviscapine	1.High-fat or high-cholesterol diet-fed mouse models 2.Diet-fed mouse models of methionine and choline deficiency	Primary hepatic stellate cell	Inhibition of the MAPK and TGF-β signaling pathways	Col1a1, Col3a1↓, CTGF↓, TIMP-1↓, TAK1↓, JNK↓, P38↓	(111)
Natural compounds and extracts	Periplaneta americana extract	Pig serum-induced liver fibrosis mice	―	Inhibition of the TGF-β1 and NF-κB signaling pathways	HA↓, LN↓, PC-III↓, IV-C↓, TGF-β↓, TIMP-1 ↓, NF-kB↓, α-SMA↓	(112)
Natural compounds and extracts	Auranofin	Bile duct ligation-induced mice model	LX2 hHSC	Inhibition of the TGF-β1, NF-κB and IκBα signaling pathways	TGF-β1↓, Col1a↓, THBS↓, FN-1↓, ET-1↓,	(113)
Natural compounds and extracts	Ferulic acid	Concanavalin-induced mouse model	―	1.Promotion of the Nrf2 signaling pathway 2.Inhibition of the TGF-β/smad3 and NF-κB signaling pathways	NF-kB↓, COX-2↓, TGF-β↓, Smad3↓, HYP↓, Caspase-3↓	(114)
Natural compounds and extracts	Echinacoside	Thioacetamide-induced mice model	―	Inhibition of the TGF-β1 signaling pathway	TGF-β1↓, β-catenin↓, SMAD4↓, MMP-9↓, PI3K↓, mTOR↓, CCN2↓, PDGFB ↓, Fascin↓, E-cadherin↑	(115)

#### 3.1.2 Small molecule TGF-β receptor antagonists

Small molecule TGF-β receptor antagonists target TGF-β receptors to prevent receptor activation, thereby regulating Smad and non-Smad signaling pathways and influencing the progression of liver fibrosis (**Table 1**). AZ12601011 (a TGF-βR I inhibitor) directly binds to TGF-βR I, blocking Smad3 activation, inhibiting the TGF-β/Smad3 signaling pathway, and alleviating paraquat-induced liver fibrosis in mice (83). AMP-activated protein kinase 5 (ARK5) prevents the degradation of TGF-βR I and maternal Smad4 proteins by inhibiting the expression of Smad ubiquitin regulatory factor 2, thereby maintaining TGF-β signal transduction (84, 85), whereas the selective inhibitor HTH-01–015 can target ARK5, reducing carbon tetrachloride (CCl4)-induced liver fibrosis deposition in mice and the expression of fibrosis-related proteins such as α-SMA and type I collagen (Col-1) (86). N-butyl-fluoperazine iodide inhibits TGF-βR II, reducing the response of HSCs to TGF-β1, decreasing TGF-β/Smad signaling transduction and fibrotic gene expression, improving CCl4- or thioacetamide-induced liver fibrosis in mice in a dose-dependent manner, and alleviating liver function damage and tissue lesions (87). J-1048 (an ALK5 inhibitor, another name for a TGF-βR I inhibitor) reduces the phosphorylation levels of TGF-βR I and Smad2/3 and increases Smad7 expression in a dose-dependent manner, thereby regulating TGF-β signal transduction and inhibiting thioacetamide-induced liver fibrosis in mice (88). Galunisertib, an ALK5 inhibitor, exerts antifibrotic effects by targeting ALK5 downstream of TGF-β signaling, thereby impeding the expression of Smad2/3-regulated genes. *In vivo*, it reduces hepatic collagen deposition, inhibits HSCs activation, prevents steatosis, and restores proper liver lobular architecture in CCl4-induced liver fibrosis rat models (89–91). J-1155 and J-115, novel thiazole derivatives that selectively target and inhibit ALK5, effectively alleviate thioacetamide-induced liver fibrosis and associated inflammation in mice through dual inhibition of the TGF-β/Smad signaling pathway and blockade of the P2X7R-NLRP3 inflammasome axis (92).

#### 3.1.3 Small molecule TGF-β signaling inhibitors

Various small molecule inhibitors exert antifibrotic effects by targeting the TGF-β signaling pathway and its key proteins (**Table 1**). Studies have shown that rsodeoxycholic acid-amino pyrimidine hybrids (12a and 12h) significantly inhibit the migration of the human hepatic stellate cell line LX2 (HSC-LX2) by blocking the TGF-β/Smad signaling pathway (93). *In vivo* studies have demonstrated that Z-RIPΔ, a novel TGF-βR I mimetic peptide, specifically binds to TGF-β1-activated HSCs, inhibiting cell proliferation and migration while reducing the expression of the fibrosis markers α-SMA and fibronectin and the TGF-β1 pathway effectors phosphorylated-Smad2/3 and phosphorylated-P38 MAPK (94). Sirtuin 6 (SIRT6) and Sirtuin 7 (SIRT7) inhibit Smad2/3 transcriptional activity by inducing its deacetylation, thereby reducing HSCs activation (95, 96).

Arbidol reduces the mRNA expression levels of PDGDR, TGF-βR I, TGF-βR II, MMP-2, and MMP-9, inhibits the phosphorylation of Smad2/3 in TGF-β1-treated HSCs and bile duct ligation-induced mice, and ultimately alleviates collagen deposition, liver injury, and fibrosis (97). G protein-coupled receptor 65 (GPR65) is closely related to liver inflammation, injury, and fibrosis. The GPR65 inhibitor ZINC62678696 reduces the activation of HSCs and hepatocyte injury induced by bile duct ligation and CCl4 by inhibiting the c-Jun N-terminal kinase (JNK) and NF-κB pathways, thereby inducing downregulation of pro-inflammatory cytokines such as TNF-α, IL-6, and TGF-β (98). Empagliflozin, a sodium-glucose cotransporter 2 inhibitor, inhibits the TGF-β pathway in HSCs by downregulating miR-34a-5p expression and upregulating Gremlin 2 in the LX-2 human hepatic stellate cell line (LX-2 HSCs) and ob/ob mouse fibrosis models, thereby improving NAFLD-related fibrosis (99).

#### 3.1.4 Natural compounds and extracts

Studies have shown that certain natural compounds and extracts inhibit CCl4-induced liver fibrosis in mice by regulating TGF-β/Smad and associated signaling pathways (**Table 1**). *In vivo* investigations demonstrate that notoginsenoside R1 (NR1), an extract derived from the traditional Chinese medicine *Panax notoginseng*, suppresses TGF-β1/Smads signaling by upregulating peroxisome proliferator-activated receptor gamma (PPAR-γ), thereby attenuating the activation of HSCs (100). Curcumin, a natural polyphenolic pigment extracted from *Curcuma longa*, exerts anti-fibrotic effects by inhibiting autophagy and inducing apoptosis in HSC-LX2 cells via activation of the PI3K/Akt/mTOR signaling pathway. Additionally, curcumin interacts with fibrosis-related proteins such as platelet-derived growth factor receptor B (PDGFRB), TIMP-1, TLR-9, and TGF-β, thereby blocking the TGF-β/Smad signaling pathway (101–104). Dihydrokaempferol (CAS 480-20-6), a natural compound related to flavonol in E. alatus, exerts its effects by inhibiting the PARP-1-regulated TGF-β1 pathway and TNF-α transcription, leading to reduced phosphorylation levels of Smad2/3 and ERK1 (105). Raspberry unripe fruit extract attenuates HSCs activation and proliferation by suppressing the TGF-β/Smads signaling pathway and downregulating the expression of related proteins, including TGF-β1, phosphorylated-Smad2/3, and Smad4 (106). Sauchinone, an active lignan found in *Saururus chinensis*, exerts anti-fibrotic effects *in vivo* by blocking TGF-β1-induced Smad2/3 phosphorylation and inhibiting the transcription of plasminogen activator inhibitor-1 (PAI-1) and MMP-2 (107). Codonopsis pilosula root polysaccharide (CPP-A-1), extracted from the traditional Chinese medicine *Radix Codonopsis*, downregulates Col-1 and α-SMA expression through the inhibition of the TLR-4/NF-κB and TGF-β1/Smad3 signaling pathways. Additionally, while also inhibits ECM production by restoring the balance between MMPs and TIMPs (108). A peptidoglycan isolated from the fruit of *Lycium barbarum*, designated as LBPW, upregulates Smad7—a negative regulator of the TGF-β/Smad signaling pathway—to delay the activation of HSCs (109). These studies collectively demonstrate that natural compounds and extracts exert antifibrotic effects in CCl4-induced liver fibrosis models by modulating the TGF-β/Smad pathway.

Esculetin, a coumarin compound belonging to the benzopyran derivative class, alleviates high-fat diet-induced liver fibrosis by activating Forkhead box O1 (FoxO1) through modulation of the Akt/PI3K/FoxO1 signaling pathway, thereby inhibiting TGF-β-mediated lipid peroxidation and ECM protein accumulation (110). Breviscapine, a flavonoid extract derived from the traditional Chinese herb *Erigeron breviscapus (Vant.)*, mitigates lipid accumulation, inflammatory cell infiltration, liver injury, and fibrosis by directly binding to transforming growth factor-β-activated kinase 1 (TAK1) and inhibiting both MAPK and TGF-β signaling pathways (111). *Periplaneta americana* extract reduces hepatic collagen deposition and reverses liver fibrosis *in vivo* by inhibiting the expression of TGF-β1, NF-κB, TIMP-1, and α-SMA (112). Auranofin, a gold-based compound, reduces hepatic steatosis and fibrosis *in vivo* by decreasing TGF-β1-induced NF-κB and inhibitor of nuclear factor kappa B alpha (IκBα) levels (113). Ferulic acid, a naturally occurring phenolic acid compound, mitigates concanavalin-induced liver fibrosis by stimulating the Nrf2 signaling pathway while inhibiting NF-κB and the TGF-β/Smad3 signaling pathway (114). Echinacoside, a natural phenol belonging to the phenylpropanoid class of caffeic acid glycosides, suppresses liver fibrosis *in vivo* by reducing the expression of TGF-β1, β-catenin, Smad4, MMP-9, PI3K, mTOR, cellular communication network factor 2 (CCN2), platelet-derived growth factor-B (PDGFB) (115).

### 3.2 Clinical applications of TGF-β inhibitors

Several TGF-β inhibitors have been evaluated in clinical trials (**Table 2**). Pirfenidone (PFD), a broad-spectrum antifibrotic drug acting on the TGF-β target, blocks TGF-β signaling through dual action pathways. On one hand, it directly binds to TGF-β1 mRNA to inhibit its transcription; on the other hand, it suppresses Smad2/3 phosphorylation and blocks their nuclear translocation. These mechanisms collectively downregulate the mRNA and protein levels of TGF-β1, TGF-βR I, and TGF-βR II, ultimately inhibiting TGF-β-induced fibroblast proliferation, ECM synthesis, and the expression of fibrotic genes (116). A single-dose open-label study by a Mexican team demonstrated that in cirrhotic patients (8 cases each with Child-Pugh class A/B), oral administration of 1200 mg prolonged-release pirfenidone (PR-PFD) resulted in the area under the curve (AUC0-last and AUC0-α) and maximal concentration 3.6-fold and 4.4-fold higher than those in healthy controls, respectively—with more pronounced increases in Child-Pugh B patients—while maintaining good tolerability (117). Another multicenter study involving 122 patients with alcoholic liver fibrosis showed that 35% of those in the PR-PFD treatment group exhibited significant fibrosis reduction (vs. 4.1% in the control group), with a 29.7% improvement rate in Child-Pugh scores (118). Hydronidone, a novel structural modification of PFD designed to reduce hepatotoxicity, was tested in a Chinese Phase II double-blind trial: chronic hepatitis B patients receiving combination therapy of hydronidone (270 mg/day) and entecavir for 52 weeks showed the most significant histological improvement in liver fibrosis with favorable safety (119). Collectively, these three studies confirm the antifibrotic potential of PR-PFD and its derivatives, with efficacy demonstrating dose-dependent trends. However, PFD is associated with inherent limitations, including high-dose-specific toxic effects and off-target reactions, which restrict its broader clinical implementation (120, 121).

**Table 2:** Clinical application of TGF-β inhibitors.

Drug	ClinicalTrial.gov Identifier	Phase	Sample size	Mechanism	Notes	Key efficacy data	Adverse events	References
PR-PFD	EI/064, 153300CT190290/2015	Phase I	N=24 (8 controls without hepatic fibrosis, 8 patients with Child-Pugh A cirrhosis, and 8 patients with Child-Pugh B cirrhosis)	Inhibition of TGF-β production	1200mg daily, blood samples were drawn at 0 hours post-dose and at 0.5, 1, 2, 2.5, 3, 3.5, 4, 4.5, 5, 6, 8, 12, 24, 30, and 36 hours	In Child - Pugh A and B, pirfenidone exposure was 3.6 - and 4.4 - fold higher. Cmax was 1.6 - and 1.8 - fold higher than in non - hepatic fibrosis group.	Nausea (20%), vomiting (12.5%),urinary tract infection (8%), transient hypertension (4%), transient ALT elevation (4%), and transient azotemia (4%).	(117)
	NCT04099407	Phase II	N = 122 (ALF, F3-F4 with Child-Pugh grade A/B)	Inhibition of TGF-β1, reduction of inflammatory cytokines (IL-6, TNF-α), endothelin-1	1200mg daily, 12 months of treatment.	Hepatic fibrosis was reversed in 35.2% of patients in the treatment group, which was higher than 4.1% in the control group (p < 0.05)	Mild nausea (9.8%), light sensitivity (7%)	(118)
Hydronidone	NCT02499562	Phase II	N = 168(CHB + cirrhosis)	Blocked TGF-β1/Smad3 pathway	52 weeks of treatment	270mg/d group: Ishak fibrosis score ↓ 1.2 ± 0.8, significantly better than placebo (p < 0.05) Fibrosis reversal rate (54.8%) was significantly higher than placebo (25.6%, p =. 006)	Thrombocytopenia (5%), mild anemia (3%)	(119)
Montelukast	NCT04080947	Phase II	N=52 (Overweight NASH patients.)	Reduce the expression of TGF-β and NF-κB in the inflammatory cell liver to reverse fibrosis.	10mg daily, 3 months of treatment.	FibroScan score(From 10.32 kPa to 7.28 kPa)	Gastrointestinal disorders, headache and sore throat(disappearance of symptoms after tolerance)	(122)

Montelukast, a leukotriene receptor antagonist, is used in the treatment of non-alcoholic steatohepatitis. In a 12-week randomized, double-blind, placebo-controlled trial conducted in Egypt, 52 overweight non-alcoholic steatohepatitis patients were randomly assigned to receive either montelukast 10 mg once daily (n=26) or placebo (n=26). Results showed significant improvements in liver stiffness, liver enzymes, metabolic parameters, TNF-α, and liver fibrosis biomarkers (hyaluronic acid and TGF-β1) in the montelukast group, with good tolerability observed (122).

Clinical trial results indicate that TGF-β inhibitors can slow or reverse liver fibrosis and improve liver function. However, their long-term safety and efficacy require further validation. Additionally, different stages and etiologies of liver fibrosis may influence the effectiveness of TGF-β inhibitors. Furthermore, hepatic and renal function can affect drug metabolism and clearance, impacting drug concentration and efficacy.

### 3.3 Combination therapy

Combination therapy has received widespread attention in liver fibrosis research, as studies have shown that different drugs can enhance antifibrotic effects through synergistic mechanisms (**Table 3**). Andrographolide, a diterpenoid compound extracted from the traditional Chinese herb *Andrographis paniculata*, has been demonstrated in preclinical studies to prevent hepatic inflammation and fibrosis (123). The combination of PFD and andrographolide inhibits the TGF-β/Smad signaling pathway, reduces Smad2/3 phosphorylation, and downregulates the expression of α-SMA, Col-1, connective tissue growth factor (CTGF), and inflammatory factors such as IL-1β, IL-6, and TNF-α. This effect suppresses HSCs activation and improves liver fibrosis (124). Curcumin 2005-8, a curcumin derivative, improves fatty liver disease through AMPK activation and autophagy regulation (125). EW-7197 (vactosertib), a small-molecule inhibitor of TGF-βR I, alleviates fibrosis by reducing reactive oxygen species via the classical Smad2/3 pathway (126). The combined application of curcumin 2005–8 and EW-7197 reduces liver fibrosis and steatohepatitis while maintaining the benefits of both drugs (127).

**Table 3:** The mechanism of combination therapy.

Drug	Animal model types	Cell types	Mechanism	Molecular expression alterations	References
PFD+AGD	Bile duct ligation-induced mice model	LX2 hHSC	Inhibition of the TGF-β/Smad signaling pathway	P-Smad2/3↓, TGF-β1↓, Col-1↓, Col-2↓, α-SMA↓, CTGF↓, IL-1β↓, IL-6↓, TNF-α↓	(123, 124)
Cur5-8+ EW-7197	Model mice on amethionine-choline deficient diet.	1.LX2 hHSC 2.AML12	1.Promotion of the AMPK signaling pathway 2.Inhibition of the Smad2/3 signaling pathway	α-SMA↓, Col-1↓, P-Smad2/3↓, Rock1↓, Srebp1c↓, AMPK↑, Nrf2↓, HO-1↓, HYP↓	(125–127)
MDB5+Anti-miR-96	Alcohol-fed mice	AKL12	Inhibition of the Hedgehog and TGF-β1 signaling pathways	TGF-β1↓, Smad7↑, Gli1↓, Col-1↓, PKA↓, ALT↓, AST↓, ECM↓, FOXO3↑	(128)
Simvastatin+NS-398	Thioacetamide-induced mice model	1.LX2 hHSC 2.Hepa RG cell	1.Promotion of the ERK1/2 and Bax/Bcl-2 signaling pathways 2.Inhibition of the TGF-βsignaling pathway	TGF-β↓, α-SMA↓, Col-1↓, P-Smad2/3↓, P-ERK1/2↑, Caspase-3↑, Bax↑, Bcl-2↓, TIMP-1/2↓, MMP1/13↓	(129)

MDB5, a small molecule inhibitor targeting the Hedgehog pathway, blocks HSCs activation by inhibiting Gli1 transcriptional activity. Lipid nanoparticles co-loaded with MDB5 and anti-miR-96 suppress the Hedgehog pathway, reducing HSCs activation and ECM gene expression while upregulating forkhead box O3 (FOXO3) and Smad7. These actions collectively inhibit TGF-β1 signal transduction and collagen synthesis, reducing liver fibrosis (128). NS-398, a selective cyclooxygenase-2 (COX-2) inhibitor, alleviates inflammation-driven fibrosis by inhibiting the synthesis of prostaglandin E2. The combination of simvastatin and NS-398 exerts antifibrotic effects by activating the ERK1/2 and Bax/Bcl-2 signaling pathways, inhibiting the TGF-β pathway, and reducing TIMP-1 and TIMP-2 expression. This effect leads to decreased liver fibrosis and collagen deposition, ultimately suppressing HSCs activation (129).

These findings suggest that the rational development of multiple antifibrotic therapies, such as the combination of TGF-β inhibitors with natural compounds, has greater therapeutic potential for improving liver fibrosis.

### 3.4 Applications of TGF-β inhibitors in liver cancer treatment

In addition to treating liver fibrosis, inhibiting the TGF-β signaling pathway helps reduce tumor progression and metastasis. A nanoparticle-based drug (NCG) encapsulating the TGF-β receptor inhibitor galunisertib and the sonosensitizer chlorin e6 has been shown to inhibit the differentiation of myeloid-derived suppressor cells, induce M1-like polarization of tumor-associated macrophages, and disrupt the immunosuppressive barrier formed by tumor-associated fibroblasts. In a mouse model of colorectal cancer liver metastasis, combination therapy with NCG (+) and anti-PD-L1 effectively inhibited colorectal cancer liver metastasis (130). The C-C motif chemokine receptor 4 inhibitor C-021 or the TGF-βR I inhibitor galunisertib, when combined with anti-PD-L1 therapy, has been found to suppress SOX12-mediated HCC progression and metastasis (131). Similarly, the TGF-βR I inhibitor (vactosertib) or the C-X-C chemokine receptor 4 inhibitor (AMD3100), in combination with anti-PD-L1, has been shown to significantly inhibit SYR-related high-mobility group box 18-mediated HCC progression and metastasis (82). Moreover, microwave ablation in combination with the ALK5 inhibitor SB-525334 effectively inactivates the TGF-β1/Smad2/Smad3 pathway, reducing the survival rate of HCC cells and promoting apoptosis (132).

## 4 Conclusion and future prospects

The TGF-β signaling pathway plays a central role in the occurrence and progression of liver fibrosis. The processes it mediates, including HSCs activation, ECM deposition, and inflammation regulation, are key mechanisms driving fibrosis. In recent years, TGF-β inhibitors have emerged as potential antifibrotic therapeutic strategies, and significant progress has been made. Various interventions, including TGF-β neutralizing antibodies, TGF-β receptor antagonists, small-molecule inhibitors, and natural compounds, have been found to inhibit HSCs activation and reduce fibrosis marker expression in both *in vitro* cell experiments and animal models. Furthermore, several TGF-β inhibitors have entered clinical trials, with certain drugs (such as PFD and galunisertib) showing promising effects in improving liver fibrosis and liver function.

Although progress has been made in the use of TGF-β inhibitors for treating various fibrosis-related diseases (133–135), the multifaceted roles of TGF-β suggest that single-target inhibition may not fully address the complexities of fibrosis treatment. Therefore, future research should focus on developing multitarget therapeutic strategies, personalized treatment approaches, and novel drug delivery systems. For example, the combination of TGF-β inhibitors with anti-inflammatory agents, antioxidants, or immunomodulators may enhance antifibrotic efficacy. Integrating gene editing technologies and cell therapy also holds promise for advancing liver fibrosis treatment. Additionally, further clinical trials are essential to evaluate the long-term efficacy and safety of TGF-β inhibitors.

In conclusion, the use of TGF-β inhibitors represents a promising antifibrotic therapeutic strategy, demonstrating potential in both basic research and clinical trials. TGF-β-targeted therapy may become an important approach for treating liver fibrosis and related liver diseases in the future through approaches to optimize drug design, develop combination therapies, and advance precision medicine applications.

## Glossary

ECM: Extracellular matrix
CLD: Chronic liver disease
HCC: Hepatocellular carcinoma
TGF-β: Transforming growth factor-beta
BMP: Bone morphogenetic protein
GDF: Growth and differentiation factor
HSCs: Hepatic stellate cells
MFBs: Myofibroblasts
MMP: Matrix metalloproteinase
TGF-βR I: Transforming growth factor beta receptor I
TGF-βR II: Transforming growth factor beta receptor II
Smad: Small mothers against decapentaplegic
R-Smad: Receptor-regulated small mothers against decapentaplegic
Co-Smad: Common small mothers against decapentaplegic
I-Smad: Inhibitory small mothers against decapentaplegic
MAPK: Mitogen-activated protein kinase
PI3K/Akt: Phosphoinositide 3-kinase/protein kinase B
NF-κB: Nuclear factor kappa-light-chain-enhancer of activated B cells
Wnt/β-catenin: Wingless-related integration site/β-catenin
mTOR: Mammalian target of rapamycin
NAFLD: Nonalcoholic fatty liver disease
TIMP: Tissue inhibitors of metalloproteinase
α-SMA: α-smooth muscle actin
CCL2: C-C motif chemokine ligand 2
TNF-α: Tumor necrosis factor-alpha
IL-6: Interleukin-6
HepG2 cell: Human hepatocellular carcinoma cell line HepG2
hHSCs: Human hepatic stellate cells
PD-L1: Programmed death-ligand 1
ARK5: AMP-activated protein kinase 5
CCl4: Carbon tetrachloride
Col-1: Type I collagen
HSC-LX2: Hepatic stellate cell line LX2
GPR65: G protein-coupled receptor 65
JNK: C-Jun N-terminal kinase
LX-2 HSCs: LX-2 human hepatic stellate cell line
NR1: Notoginsenoside R1
PPAR-γ: Peroxisome proliferator-activated receptor gamma
PDGFRB: Platelet-derived growth factor receptor B
PAI-1: Plasminogen activator inhibitor-1
CPP-A-1: Codonopsis pilosula root polysaccharide
TAK1: Transforming growth factor-β-activated kinase 1
IκBα: Inhibitor of nuclear factor kappa B alpha
CCN2: Cellular communication network factor 2
PDGFB: Platelet-derived growth factor B
TLR: Toll-like receptor
FoxO1: Forkhead box O1
PFD: Pirfenidone
PR-PFD: Prolonged-release pirfenidone formulation
CTGF: Connective tissue growth factor
COX-2: Cyclooxygenase-2
NCG: Nanoparticle-based drug

## References

[1] Iredale JP. Models of liver fibrosis: exploring the dynamic nature of inflammation and repair in a solid organ. J Clin Invest. (2007) 117:539–48. doi: 10.1172/JCI30542 PMC1804370 17332881

[2] Ekstedt M Franzén LE Mathiesen UL Thorelius L Holmqvist M Bodemar G. Long-term follow-up of patients with NAFLD and elevated liver enzymes. Hepatology. (2006) 44:865–73. doi: 10.1002/hep.21327 17006923

[3] Cheemerla S Balakrishnan M. Global epidemiology of chronic liver disease. Clin Liver Dis. (2021) 17:365–70. doi: 10.1002/cld.1061 PMC8177826 34136143

[4] Asrani SK Devarbhavi H Eaton J Kamath PS. Burden of liver diseases in the world. J Hepatol. (2019) 70:151–71. doi: 10.1016/j.jhep.2018.09.014 30266282

[5] Tapper EB Parikh ND. Mortality due to cirrhosis and liver cancer in the United States, 1999-2016: observational study. BMJ. (2018) 362:k2817. doi: 10.1136/bmj.k2817 30021785 PMC6050518

[6] GBD 2017 Disease and Injury Incidence and Prevalence Collaborators. Global, regional, and national incidence, prevalence, and years lived with disability for 354 diseases and injuries for 195 countries and territories, 1990-2017: a systematic analysis for the Global Burden of Disease Study 2017. Lancet. (2018) 392:1789–858. doi: 10.1016/S0140-6736(18)32279-7 PMC6227754 30496104

[7] David CJ Massagué J. Contextual determinants of TGF-β action in development, immunity and cancer. Nat Rev Mol Cell Biol. (2018) 19:419–35. doi: 10.1038/s41580-018-0007-0 PMC7457231 29643418

[8] Lan HY. Diverse roles of TGF-β/Smads in renal fibrosis and inflammation. Int J Biol Sci. (2011) 7:1056–67. doi: 10.7150/ijbs.7.1056 PMC3174390 21927575

[9] Cao M Zhao Q Xia H Lyu S Luo J Fu K. Intracellular and extracellular Cyclophilin a promote cardiac fibrosis through TGF-β signaling in response to angiotensin II. Biochem Pharmacol. (2024) 225:116271. doi: 10.1016/j.bcp.2024.116271 38723722

[10] Wang Y Ping Z Gao H Liu Z Xv Q Jiang X. LYC inhibits the AKT signaling pathway to activate autophagy and ameliorate TGFB-induced renal fibrosis. Autophagy. (2024) 20:1114–33. doi: 10.1080/15548627.2023.2287930 PMC11135866 38037248

[11] Trinh-Minh T Chen CW Tran Manh C Li YN Zhu H Zhou X. Noncanonical WNT5A controls the activation of latent TGF-β to drive fibroblast activation and tissue fibrosis. J Clin Invest. (2024) 134:e159884. doi: 10.1172/JCI159884 38747285 PMC11093613

[12] Humeres C Shinde AV Tuleta I Hernandez SC Hanna A Huang S. Fibroblast Smad7 induction protects the remodeling pressure-overloaded heart. Circ Res. (2024) 135:453–69. doi: 10.1161/CIRCRESAHA.123.323360 PMC11257802 38899461

[13] Sakai K Jawaid S Sasaki T Bou-Gharios G Sakai T. Transforming growth factor-β–independent role of connective tissue growth factor in the development of liver fibrosis. Am J Pathol. (2014) 184:2611–7. doi: 10.1016/j.ajpath.2014.06.009 PMC4715221 25108224

[14] Fabregat I Caballero-Díaz D. Transforming growth factor-β-induced cell plasticity in liver fibrosis and hepatocarcinogenesis. Front Oncol. (2018) 8:357. doi: 10.3389/fonc.2018.00357 30250825 PMC6139328

[15] Fu X Qie J Fu Q Chen J Jin Y Ding Z. miR-20a-5p/TGFBR2 axis affects pro-inflammatory macrophages and aggravates liver fibrosis. Front Oncol. (2020) 10:107. doi: 10.3389/fonc.2020.00107 32117757 PMC7031347

[16] Tsuchida T Friedman SL. Mechanisms of hepatic stellate cell activation. Nat Rev Gastroenterol Hepatol. (2017) 14:397–411. doi: 10.1038/nrgastro.2017.38 28487545

[17] Kuwahara F Kai H Tokuda K Kai M Takeshita A Egashira K. Transforming growth factor-beta function blocking prevents myocardial fibrosis and diastolic dysfunction in pressure-overloaded rats. Circulation. (2002) 106:130–5. doi: 10.1161/01.cir.0000020689.12472.e0 12093782

[18] Meng L Lu Y Wang X Cheng C Xue F Xie L. NPRC deletion attenuates cardiac fibrosis in diabetic mice by activating PKA/PKG and inhibiting TGF-β1/Smad pathways. Sci Adv. (2023) 9:eadd4222. doi: 10.1126/sciadv.add4222 37531438 PMC10396312

[19] Qiu Y Song X Liu Y Wu Y Shi J Zhang F. Application of recombinant TGF-β1 inhibitory peptide to alleviate isoproterenol-induced cardiac fibrosis. Appl Microbiol Biotechnol. (2023) 107:6251–62. doi: 10.1007/s00253-023-12722-x 37606791

[20] Fukasawa H Yamamoto T Suzuki H Togawa A Ohashi N Fujigaki Y. Treatment with anti-TGF-beta antibody ameliorates chronic progressive nephritis by inhibiting Smad/TGF-beta signaling. Kidney Int. (2004) 65:63–74. doi: 10.1111/j.1523-1755.2004.00393.x 14675037

[21] Liang X Schnaper HW Matsusaka T Pastan I Ledbetter S Hayashida T. Anti-TGF-β antibody, 1D11, ameliorates glomerular fibrosis in mouse models after the onset of proteinuria. PloS One. (2016) 11:e0155534. doi: 10.1371/journal.pone.0155534 27187580 PMC4871338

[22] McGaraughty S Davis-Taber RA Zhu CZ Cole TB Nikkel AL Chhaya M. Targeting anti–TGF-β therapy to fibrotic kidneys with a dual specificity antibody approach. J Am Soc Nephrol. (2017) 28:3616–26. doi: 10.1681/ASN.2017010013 PMC5698069 28827403

[23] Hinck AP Mueller TD Springer TA. Structural biology and evolution of the TGF-β family. Cold Spring Harb Perspect Biol. (2016) 8:a022103. doi: 10.1101/cshperspect.a022103 27638177 PMC5131774

[24] Dong X Zhao B Iacob RE Zhu J Koksal AC Lu C. Force interacts with macromolecular structure in activation of TGF-β. Nature. (2017) 542:55–9. doi: 10.1038/nature21035 PMC5586147 28117447

[25] Clark DA Coker R. Molecules in focus tansforming growth factor-beta (TGF-*β*). Int J Biochem Cell Biol. (1998) 30:293–8. doi: 10.1016/S1357-2725(97)00128-3 9611771

[26] Chang Y Bach L Hasiuk M Wen L Elmzzahi T Tsui C. TGF-β specifies TFH versus TH17 cell fates in murine CD4+ T cells through c-Maf. Sci Immunol. (2024) 9:eadd4818. doi: 10.1126/sciimmunol.add4818 38427718

[27] Haider S Lackner AI Dietrich B Kunihs V Haslinger P Meinhardt G. Transforming growth factor-β signaling governs the differentiation program of extravillous trophoblasts in the developing human placenta. Proc Natl Acad Sci USA. (2022) 119:e2120667119. doi: 10.1073/pnas.2120667119 35867736 PMC9282384

[28] Tsukui T Wolters PJ Sheppard D. Alveolar fibroblast lineage orchestrates lung inflammation and fibrosis. Nature. (2024) 631:627–34. doi: 10.1038/s41586-024-07660-1 PMC12088911 38987592

[29] Geng K Ma X Jiang Z Gu J Huang W Wang W. WDR74 facilitates TGF-β/Smad pathway activation to promote M2 macrophage polarization and diabetic foot ulcer wound healing in mice. Cell Biol Toxicol. (2023) 39:1577–91. doi: 10.1007/s10565-022-09748-8 35982296

[30] Chen W. TGF-β regulation of T cells. Annu Rev Immunol. (2023) 41:483–512. doi: 10.1146/annurev-immunol-101921-045939 36750317 PMC12453633

[31] Moreau JM Velegraki M Bolyard C Rosenblum MD Li Z. Transforming growth factor–β1 in regulatory T cell biology. Sci Immunol. (2022) 7:eabi4613. doi: 10.1126/sciimmunol.abi4613 35302863 PMC10552796

[32] Chang C. Agonists and antagonists of TGF-β family ligands. Cold Spring Harb Perspect Biol. (2016) 8:a021923. doi: 10.1101/cshperspect.a021923 27413100 PMC4968162

[33] Morikawa M Derynck R Miyazono K. TGF-β and the TGF-β family: context-dependent roles in cell and tissue physiology. Cold Spring Harb Perspect Biol. (2016) 8:a021873. doi: 10.1101/cshperspect.a021873 27141051 PMC4852809

[34] Frangogiannis NG. Transforming growth factor–β in tissue fibrosis. J Exp Med. (2020) 217:e20190103. doi: 10.1084/jem.20190103 32997468 PMC7062524

[35] Blakytny R Ludlow A Martin GEM Ireland G Lund LR Ferguson MWJ. Latent TGF-β1 activation by platelets. J Cell Physiol. (2004) 199:67–76. doi: 10.1002/jcp.10454 14978736

[36] Huang H Wang Z Zhang Y Pradhan RN Ganguly D Chandra R. Mesothelial cell-derived antigen-presenting cancer-associated fibroblasts induce expansion of regulatory T cells in pancreatic cancer. Cancer Cell. (2022) 40:656–673.e7. doi: 10.1016/j.ccell.2022.04.011 35523176 PMC9197998

[37] Frangogiannis NG. TGF-β as a therapeutic target in the infarcted and failing heart: cellular mechanisms, challenges, and opportunities. Expert Opin Ther Targets. (2024) 28:45–56. doi: 10.1080/14728222.2024.2316735 38329809

[38] Miyazawa K Shinozaki M Hara T Furuya T Miyazono K. Two major Smad pathways in TGF-beta superfamily signalling. Genes Cells. (2002) 7:1191–204. doi: 10.1046/j.1365-2443.2002.00599.x 12485160

[39] Dewidar B Meyer C Dooley S Meindl-Beinker N. TGF-β in hepatic stellate cell activation and liver fibrogenesis—updated 2019. Cells. (2019) 8:1419. doi: 10.3390/cells8111419 31718044 PMC6912224

[40] Chanda D Otoupalova E Smith SR Volckaert T De Langhe SP Thannickal VJ. Developmental pathways in the pathogenesis of lung fibrosis. Mol Aspects Med. (2019) 65:56–69. doi: 10.1016/j.mam.2018.08.004 30130563 PMC6374163

[41] Isaka Y. Targeting TGF-β signaling in kidney fibrosis. Int J Mol Sci. (2018) 19:2532. doi: 10.3390/ijms19092532 30150520 PMC6165001

[42] Principe DR Doll JA Bauer J Jung B Munshi HG Bartholin L. TGF-β: duality of function between tumor prevention and carcinogenesis. J Natl Cancer Inst. (2014) 106:djt369. doi: 10.1093/jnci/djt369 24511106 PMC3952197

[43] Lebrun JJ. The dual role of TGFβ in human cancer: from tumor suppression to cancer metastasis. ISRN Mol Biol. (2012) 2012:381428. doi: 10.5402/2012/381428 27340590 PMC4899619

[44] Wrana JL Attisano L Wieser R Ventura F Massagué J. Mechanism of activation of the TGF-beta receptor. Nature. (1994) 370:341–7. doi: 10.1038/370341a0 8047140

[45] Kawabata M Miyazono K. Signal transduction of the TGF-beta superfamily by Smad proteins. J Biochem. (1999) 125:9–16. doi: 10.1093/oxfordjournals.jbchem.a022273 9880789

[46] Chen SJ Yuan W Lo S Trojanowska M Varga J. Interaction of smad3 with a proximal smad-binding element of the human alpha2(I) procollagen gene promoter required for transcriptional activation by TGF-beta. J Cell Physiol. (2000) 183:381–92. doi: 10.1002/(SICI)1097-4652(200006)183:3<381::AID-JCP11>3.0.CO;2-O 10797313

[47] Nakao A Imamura T Souchelnytskyi S Kawabata M Ishisaki A Oeda E. TGF-beta receptor-mediated signalling through Smad2, Smad3 and Smad4. EMBO J. (1997) 16:5353–62. doi: 10.1093/emboj/16.17.5353 PMC1170167 9311995

[48] Shi Y Massagué J. Mechanisms of TGF-beta signaling from cell membrane to the nucleus. Cell. (2003) 113:685–700. doi: 10.1016/s0092-8674(03)00432-x 12809600

[49] Retting KN Song B Yoon BS Lyons KM. BMP canonical Smad signaling through Smad1 and Smad5 is required for endochondral bone formation. Development. (2009) 136:1093–104. doi: 10.1242/dev.029926 PMC2668702 19224984

[50] Wang B Zhao Q Gong X Wang C Bai Y Wang H. Transmembrane anterior posterior transformation 1 regulates BMP signaling and modulates the protein stability of SMAD1/5. J Biol Chem. (2022) 298:102684. doi: 10.1016/j.jbc.2022.102684 36370851 PMC9763856

[51] Imamura T Takase M Nishihara A Oeda E Hanai J Kawabata M. Smad6 inhibits signalling by the TGF-beta superfamily. Nature. (1997) 389:622–6. doi: 10.1038/39355 9335505

[52] Mochizuki T Miyazaki H Hara T Furuya T Imamura T Watabe T. Roles for the MH2 domain of Smad7 in the specific inhibition of transforming growth factor-β superfamily signaling. J Biol Chem. (2004) 279:31568–74. doi: 10.1074/jbc.M313977200 15148321

[53] Hayashi H Abdollah S Qiu Y Cai J Xu YY Grinnell BW. The MAD-related protein smad7 associates with the TGFβ Receptor and functions as an antagonist of TGFβ Signaling. Cell. (1997) 89:1165–73. doi: 10.1016/S0092-8674(00)80303-7 9215638

[54] Zhang YE. Non-Smad pathways in TGF-β signaling. Cell Res. (2009) 19:128–39. doi: 10.1038/cr.2008.328 PMC2635127 19114990

[55] Zhang YE. Non-smad signaling pathways of the TGF-β Family. Cold Spring Harb Perspect Biol. (2017) 9:a022129. doi: 10.1101/cshperspect.a022129 27864313 PMC5287080

[56] Luo K. Signaling cross talk between TGF-β/smad and other signaling pathways. Cold Spring Harb Perspect Biol. (2017) 9:a022137. doi: 10.1101/cshperspect.a022137 27836834 PMC5204325

[57] Zhuang C Guo Z Zhu J Wang W Sun R Qi M. PTEN inhibitor attenuates cardiac fibrosis by regulating the M2 macrophage phenotype via the PI3K/AKT/TGF-β/Smad 2/3 signaling pathway. Int J Cardiol. (2022) 356:88–96. doi: 10.1016/j.ijcard.2022.04.007 35395283

[58] Mallat A Lotersztajn S. Cellular Mechanisms of Tissue Fibrosis. 5. Novel insights into liver fibrosis. Am J Physiol Cell Physiol. (2013) 305:C789–99. doi: 10.1152/ajpcell.00230.2013 23903700

[59] Parola M Pinzani M. Liver fibrosis: Pathophysiology, pathogenetic targets and clinical issues. Mol Aspects Med. (2019) 65:37–55. doi: 10.1016/j.mam.2018.09.002 30213667

[60] Ginès P Krag A Abraldes JG Solà E Fabrellas N Kamath PS. Liver cirrhosis. Lancet. (2021) 398:1359–76. doi: 10.1016/S0140-6736(21)01374-X 34543610

[61] Kumar S Duan Q Wu R Harris EN Su Q. Pathophysiological communication between hepatocytes and non-parenchymal cells in liver injury from NAFLD to liver fibrosis. Adv Drug Delivery Rev. (2021) 176:113869. doi: 10.1016/j.addr.2021.113869 PMC11792083 34280515

[62] Bouwens L Baekeland M De Zanger R Wisse E. Quantitation, tissue distribution and proliferation kinetics of Kupffer cells in normal rat liver. Hepatology. (1986) 6:718–22. doi: 10.1002/hep.1840060430 3733004

[63] Elpek GÖ. Cellular and molecular mechanisms in the pathogenesis of liver fibrosis: An update. World J Gastroenterol. (2014) 20:7260. doi: 10.3748/wjg.v20.i23.7260 24966597 PMC4064072

[64] Blaner WS O’Byrne SM Wongsiriroj N Kluwe J D’Ambrosio D Jiang H. Hepatic stellate cell lipid droplets: a specialized lipid droplet for retinoid storage. Biochim Biophys Acta. (2009) 1791:467–73. doi: 10.1016/j.bbalip.2008.11.001 PMC2719539 19071229

[65] McConnell MJ Kostallari E Ibrahim SH Iwakiri Y. The evolving role of liver sinusoidal endothelial cells in liver health and disease. Hepatology. (2023) 78:649–69. doi: 10.1097/HEP.0000000000000207 PMC10315420 36626620

[66] Koyama Y Brenner DA. Liver inflammation and fibrosis. J Clin Invest. (2017) 127:55–64. doi: 10.1172/JCI88881 28045404 PMC5199698

[67] Ramachandran P Pellicoro A Vernon MA Boulter L Aucott RL Ali A. Differential Ly-6C expression identifies the recruited macrophage phenotype, which orchestrates the regression of murine liver fibrosis. Proc Natl Acad Sci U S A. (2012) 109:E3186–95. doi: 10.1073/pnas.1119964109 PMC3503234 23100531

[68] Tacke F Zimmermann HW. Macrophage heterogeneity in liver injury and fibrosis. J Hepatology. (2014) 60:1090–6. doi: 10.1016/j.jhep.2013.12.025 24412603

[69] Higashi T Friedman SL Hoshida Y. Hepatic stellate cells as key target in liver fibrosis. Advanced Drug delivery Rev. (2017) 121:27. doi: 10.1016/j.addr.2017.05.007 PMC5682243 28506744

[70] Xu F Liu C Zhou D Zhang L. TGF-β/Smad pathway and its regulation in hepatic fibrosis. J Histochem Cytochem. (2016) 64:157–67. doi: 10.1369/0022155415627681 PMC4810800 26747705

[71] Dooley S Hamzavi J Breitkopf K Wiercinska E Said HM Lorenzen J. Smad7 prevents activation of hepatic stellate cells and liver fibrosis in rats. Gastroenterology. (2003) 125:178–91. doi: 10.1016/S0016-5085(03)00666-8 12851882

[72] Zhang K Han X Zhang Z Zheng L Hu Z Yao Q. The liver-enriched lnc-LFAR1 promotes liver fibrosis by activating TGF-β and Notch pathways. Nat Commun. (2017) 8:144. doi: 10.1038/s41467-017-00204-4 28747678 PMC5529527

[73] Wang JN Li L Li LY Yan Q Li J Xu T. Emerging role and therapeutic implication of Wnt signaling pathways in liver fibrosis. Gene. (2018) 674:57–69. doi: 10.1016/j.gene.2018.06.053 29944952

[74] Liu H Huang H Liu Y Yang Y Deng H Wang X. Adipose-derived mesenchymal stem cells inhibit hepatic stellate cells activation to alleviate liver fibrosis via Hippo pathway. Stem Cell Res Ther. (2024) 15:378. doi: 10.1186/s13287-024-03988-7 39449061 PMC11515333

[75] Weiskirchen R Weiskirchen S Tacke F. Organ and tissue fibrosis: Molecular signals, cellular mechanisms and translational implications. Mol Aspects Med. (2019) 65:2–15. doi: 10.1016/j.mam.2018.06.003 29958900

[76] Wang C Ma C Gong L Guo Y Fu K Zhang Y. Macrophage polarization and its role in liver disease. Front Immunol. (2021) 12:803037. doi: 10.3389/fimmu.2021.803037 34970275 PMC8712501

[77] Pellicoro A Aucott RL Ramachandran P Robson AJ Fallowfield JA Snowdon VK. Elastin accumulation is regulated at the level of degradation by macrophage metalloelastase (MMP-12) during experimental liver fibrosis. Hepatology. (2012) 55:1965–75. doi: 10.1002/hep.25567 22223197

[78] Feng M Ding J Wang M Zhang J Zhu X Guan W. Kupffer-derived matrix metalloproteinase-9 contributes to liver fibrosis resolution. Int J Biol Sci. (2018) 14:1033–40. doi: 10.7150/ijbs.25589 PMC6036732 29989076

[79] Rampa DR Feng H Allur-Subramaniyan S Shim K Pekcec A Lee D. Kinin B1 receptor blockade attenuates hepatic fibrosis and portal hypertension in chronic liver diseases in mice. J Transl Med. (2022) 20:590. doi: 10.1186/s12967-022-03808-7 36514072 PMC9746183

[80] Calixto-Tlacomulco S Luna-Reyes I Delgado-Coello B Gutiérrez-Vidal R Reyes-Grajeda JP Mas-Oliva J. CETP-derived peptide seq-1, the key component of HB-ATV-8 vaccine prevents stress responses, and promotes downregulation of pro-fibrotic genes in hepatocytes and stellate cells. Arch Med Res. (2024) 55:102937. doi: 10.1016/j.arcmed.2023.102937 38301446

[81] Lan Y Yeung TL Huang H Wegener AA Saha S Toister-Achituv M. Colocalized targeting of TGF-β and PD-L1 by bintrafusp alfa elicits distinct antitumor responses. J Immunother Cancer. (2022) 10:e004122. doi: 10.1136/jitc-2021-004122 35858707 PMC9305820

[82] Chen J Feng W Sun M Huang W Wang G Chen X. TGF-β1-induced SOX18 elevation promotes hepatocellular carcinoma progression and metastasis through transcriptionally upregulating PD-L1 and CXCL12. Gastroenterology. (2024) 167:264–80. doi: 10.1053/j.gastro.2024.02.025 38417530

[83] Zhang H Yang H Liu X Ying J Zu T Jiang J. Targeted inhibition of transforming growth factor-β type I receptor by AZ12601011 improves paraquat poisoning-induced multiple organ fibrosis. Pestic Biochem Physiol. (2024) 200:105831. doi: 10.1016/j.pestbp.2024.105831 38582594

[84] Ye Z He Q Wang Q Lin Y Cen K Chen X. LINC00922 promotes the proliferation, migration, invasion and EMT process of liver cancer cells by regulating miR-424-5p/ARK5. Mol Cell Biochem. (2021) 476:3757–69. doi: 10.1007/s11010-021-04196-0 34097192

[85] Liu L Ulbrich J Müller J Wüstefeld T Aeberhard L Kress TR. Deregulated MYC expression induces dependence upon AMPK-related kinase 5. Nature. (2012) 483:608–12. doi: 10.1038/nature10927 22460906

[86] You Y Gao C Wu J Qu H Xiao Y Kang Z. Enhanced expression of ARK5 in hepatic stellate cell and hepatocyte synergistically promote liver fibrosis. Int J Mol Sci. (2022) 23:13084. doi: 10.3390/ijms232113084 36361872 PMC9655442

[87] Shen D Cheng H Cai B Cai W Wang B Zhu Q. N-n-butyl haloperidol iodide ameliorates liver fibrosis and hepatic stellate cell activation in mice. Acta Pharmacol Sin. (2022) 43:133–45. doi: 10.1038/s41401-021-00630-7 PMC8724321 33758354

[88] Yang HX Guo FY Lin YC Wu YL Nan JX Jin CH. Synthesis of and anti-fibrotic effect of pyrazole derivative J-1048: inhibition of ALK5 as a novel approach to liver fibrosis targeting inflammation. Bioorg Chem. (2023) 139:106723. doi: 10.1016/j.bioorg.2023.106723 37459824

[89] Peterson JM Jay JW Wang Y Joglar AA Prasai A Palackic A. Galunisertib exerts antifibrotic effects on TGF-β-induced fibroproliferative dermal fibroblasts. Int J Mol Sci. (2022) 23:6689. doi: 10.3390/ijms23126689 35743131 PMC9223605

[90] van Leeuwen LL Ruigrok MJR Kessler BM Leuvenink HGD Olinga P. Targeted delivery of galunisertib using machine perfusion reduces fibrogenesis in an integrated ex vivo renal transplant and fibrogenesis model. Br J Pharmacol. (2024) 181:464–79. doi: 10.1111/bph.16220 37596999

[91] Panzarini E Leporatti S Tenuzzo BA Quarta A Hanafy NAN Giannelli G. Therapeutic effect of polymeric nanomicelles formulation of LY2157299-galunisertib on CCl4-induced liver fibrosis in rats. J Pers Med. (2022) 12:1812. doi: 10.3390/jpm12111812 36579532 PMC9692463

[92] Jiang XL Liu C Zhan ZY Lan XQ Wu YL Nan JX. Thiazole isomers as potential ALK5 inhibitors alleviate P2X7R-mediated inflammation during liver fibrosis. Int Immunopharmacol. (2025) 153:114472. doi: 10.1016/j.intimp.2025.114472 40117804

[93] Hu Y Li L Tian Y Xiao Y Tang J Zeng S. Design, synthesis and evaluation of novel UDCA-aminopyrimidine hybrids as ATX inhibitors for the treatment of hepatic and pulmonary fibrosis. Eur J Med Chem. (2024) 264:116029. doi: 10.1016/j.ejmech.2023.116029 38091892

[94] Liu X Wang X Xu L Fan J Yuan Q Zhang F. Targeting delivery of a novel TGF-β type I receptor-mimicking peptide to activated hepatic stellate cells for liver fibrosis therapy via inhibiting the TGF-β1/smad and p38 MAPK signaling pathways. Eur J Pharmacol. (2024) 977:176708. doi: 10.1016/j.ejphar.2024.176708 38843945

[95] Ding C. SIRT7 protects against liver fibrosis by suppressing stellate cell activation via TGF-β/SMAD2/3 pathway. BioMed Pharmacother. (2024) 180:117477. doi: 10.1016/j.biopha.2024.117477 39316972

[96] Dong XC. Sirtuin 6—a key regulator of hepatic lipid metabolism and liver health. Cells. (2023) 12:663. doi: 10.3390/cells12040663 36831330 PMC9954390

[97] Ren Y Chen Y Tang EH Hu Y Niu B Liang H. Arbidol attenuates liver fibrosis and activation of hepatic stellate cells by blocking TGF-β1 signaling. Eur J Pharmacol. (2024) 967:176367. doi: 10.1016/j.ejphar.2024.176367 38325795

[98] Zhang K Zhang MX Meng XX Zhu J Wang JJ He YF. Targeting GPR65 alleviates hepatic inflammation and fibrosis by suppressing the JNK and NF-κB pathways. Mil Med Res. (2023) 10:56. doi: 10.1186/s40779-023-00494-4 38001521 PMC10675918

[99] Shen Y Cheng L Xu M Wang W Wan Z Xiong H. SGLT2 inhibitor empagliflozin downregulates miRNA-34a-5p and targets GREM2 to inactivate hepatic stellate cells and ameliorate non-alcoholic fatty liver disease-associated fibrosis. Metabolism. (2023) 146:155657. doi: 10.1016/j.metabol.2023.155657 37422021

[100] Guo C Lai L Ma B Huang Q Wang Z. Notoginsenoside R1 targets PPAR-γ to inhibit hepatic stellate cell activation and ameliorates liver fibrosis. Exp Cell Res. (2024) 437:113992. doi: 10.1016/j.yexcr.2024.113992 38492634

[101] Ceccherini E Signore G Tedeschi L Vozzi F Di Giorgi N Michelucci E. Proteomic modulation in TGF-β-treated cholangiocytes induced by curcumin nanoparticles. Int J Mol Sci. (2023) 24:10481. doi: 10.3390/ijms241310481 37445659 PMC10341614

[102] Gabr SA Elsaed WM Eladl MA El-Sherbiny M Ebrahim HA Asseri SM. Curcumin modulates oxidative stress, fibrosis, and apoptosis in drug-resistant cancer cell lines. Life. (2022) 12:1427. doi: 10.3390/life12091427 36143462 PMC9504331

[103] Shu Y He Y Ye G Liu X Huang J Zhang Q. Curcumin inhibits the activity and induces apoptosis of activated hepatic stellate cell by suppressing autophagy. J Cell Biochem. (2023) 124:1764–78. doi: 10.1002/jcb.30487 37909649

[104] Elzoheiry A Ayad E Omar N Elbakry K Hyder A. Anti-liver fibrosis activity of curcumin/chitosan-coated green silver nanoparticles. Sci Rep. (2022) 12:18403. doi: 10.1038/s41598-022-23276-9 36319750 PMC9626641

[105] Huang H Wei S Wu X Zhang M Zhou B Huang D. Dihydrokaempferol attenuates CCl4-induced hepatic fibrosis by inhibiting PARP-1 to affect multiple downstream pathways and cytokines. Toxicol Appl Pharmacol. (2023) 464:116438. doi: 10.1016/j.taap.2023.116438 36841340

[106] Wu J Zhang D Zhu B Wang S Xu Y Zhang C. Rubus chingii hu. unripe fruits extract ameliorates carbon tetrachloride-induced liver fibrosis and improves the associated gut microbiota imbalance. Chin Med. (2022) 17:56. doi: 10.1186/s13020-022-00607-6 35549741 PMC9097331

[107] Lee JH Jang EJ Seo HL Ku SK Lee JR Shin SS. Sauchinone attenuates liver fibrosis and hepatic stellate cell activation through TGF-β/Smad signaling pathway. Chem-Biol Interact. (2014) 224:58–67. doi: 10.1016/j.cbi.2014.10.005 25451574

[108] Meng X Kuang H Wang Q Zhang H Wang D Kang T. A polysaccharide from codonopsis pilosula roots attenuates carbon tetrachloride-induced liver fibrosis via modulation of TLR4/NF-κB and TGF-β1/Smad3 signaling pathway. Int Immunopharmacol. (2023) 119:110180. doi: 10.1016/j.intimp.2023.110180 37068337

[109] Nie YM Zhou WQ Niu T Mao MF Zhan YX Li Y. Peptidoglycan isolated from the fruit of Lycium barbarum alleviates liver fibrosis in mice by regulating the TGF-β/Smad7 signaling and gut microbiota. Acta Pharmacol Sin. (2025) 46:1329–44. doi: 10.1038/s41401-024-01454-x PMC12032012 39833303

[110] Pandey A Raj P Goru SK Kadakol A Malek V Sharma N. Esculetin ameliorates hepatic fibrosis in high fat diet induced non-alcoholic fatty liver disease by regulation of FoxO1 mediated pathway. Pharmacol Rep. (2017) 69:666–72. doi: 10.1016/j.pharep.2017.02.005 28527877

[111] Lan T Jiang S Zhang J Weng Q Yu Y Li H. Breviscapine alleviates NASH by inhibiting TGF-β-activated kinase 1-dependent signaling. Hepatology. (2022) 76:155–71. doi: 10.1002/hep.32221 PMC9299589 34717002

[112] Li D Ma D Liu Y Liu L Chen Y Liu H. Extracts of periplaneta americana alleviate hepatic fibrosis by affecting hepatic TGF-β and NF-κB expression in rats with pig serum-induced liver fibrosis. Folia Histochem Cytobiol. (2022) 60:125–35. doi: 10.5603/FHC.a2022.0011 35575220

[113] Lee SM Koh DH Jun DW Roh YJ Kang HT Oh JH. Auranofin attenuates hepatic steatosis and fibrosis in nonalcoholic fatty liver disease via NRF2 and NF- κB signaling pathways. Clin Mol Hepatol. (2022) 28:827–40. doi: 10.3350/cmh.2022.0068 PMC9597229 35730208

[114] Elazab ST Hsu WH. Ferulic acid ameliorates concanavalin a-induced hepatic fibrosis in mice via suppressing TGF-β/smad signaling. Toxicol Appl Pharmacol. (2024) 492:117099. doi: 10.1016/j.taap.2024.117099 39260469

[115] Albalawi AZ Alatawi AS Al-Atwi SM Alhwyty LS Alharbi KM Alshehri SA. EChinacoside ameliorates hepatic fibrosis and tumor invasion in rats with thioacetamide-induced hepatocellular carcinoma. Biomol Biomed. (2024) 24:1186–98. doi: 10.17305/bb.2024.10367 PMC11379005 38461536

[116] Xie Y Jiang H Zhang Q Mehrotra S Abel PW Toews ML. Upregulation of RGS2: a new mechanism for pirfenidone amelioration of pulmonary fibrosis. Respir Res. (2016) 17:103. doi: 10.1186/s12931-016-0418-4 27549302 PMC4994235

[117] Poo JL Aguilar JR Bernal-Reyes R Alonso-Campero R Gasca F Hernández L. Prolonged release pirfenidone pharmacokinetics is modified in cirrhosis GENESIS study. BioMed Pharmacother. (2023) 168:115712. doi: 10.1016/j.biopha.2023.115712 37871556

[118] Poo JL Torre A Aguilar-Ramírez JR Cruz M Mejía-Cuán L Cerda E. Benefits of prolonged-release pirfenidone plus standard of care treatment in patients with advanced liver fibrosis: PROMETEO study. Hepatol Int. (2020) 14:817–27. doi: 10.1007/s12072-020-10069-3 PMC7561536 32813194

[119] Cai X Liu X Xie W Ma A Tan Y Shang J. Hydronidone for the treatment of liver fibrosis related to chronic hepatitis B: A phase 2 randomized controlled trial. Clin Gastroenterol Hepatol. (2023) 21:1893–1901.e7. doi: 10.1016/j.cgh.2022.05.056 35842120

[120] Kuronuma K Susai N Kuroita T Yamamoto H Yoshioka T Kaneko S. Analysis of real-world data and a mouse model indicates that pirfenidone causes pellagra. ERJ Open Res. (2022) 8:00245–2022. doi: 10.1183/23120541.00245-2022 PMC9589320 36299372

[121] Cottin V Koschel D Günther A Albera C Azuma A Sköld CM. Long-term safety of pirfenidone: results of the prospective, observational PASSPORT study. ERJ Open Res. (2018) 4:00084–2018. doi: 10.1183/23120541.00084-2018 PMC6194203 30364407

[122] Abdallah MS Eldeen AH Tantawy SS Mostafa TM. The leukotriene receptor antagonist montelukast in the treatment of non-alcoholic steatohepatitis: A proof-of-concept, randomized, double-blind, placebo-controlled trial. Eur J Pharmacol. (2021) 906:174295. doi: 10.1016/j.ejphar.2021.174295 34214585

[123] Lin L Li R Cai M Huang J Huang W Guo Y. Andrographolide ameliorates liver fibrosis in mice: involvement of TLR4/NF-κB and TGF-β1/smad2 signaling pathways. Oxid Med Cell Longev. (2018) 2018:7808656. doi: 10.1155/2018/7808656 29743985 PMC5878918

[124] Xu G Ma T Zhou C Zhao F Peng K Li B. Combination of pirfenidone and andrographolide ameliorates hepatic stellate cell activation and liver fibrosis by mediating TGF- *β*/smad signaling pathway. Anal Cell Pathol. (2024) 2024:2751280. doi: 10.1155/2024/2751280 PMC11213636 38946862

[125] Lee ES Kwon MH Kim HM Woo HB Ahn CM Chung CH. Curcumin analog CUR5–8 ameliorates nonalcoholic fatty liver disease in mice with high-fat diet-induced obesity. Metabolism. (2020) 103:154015. doi: 10.1016/j.metabol.2019.154015 31758951

[126] Kim BG Choi SH Luo G Sergeeva O Lee Z Driscoll J. Vactosertib, a TGF-ß receptor I kinase/ALK5 inhibitor, diminishes tumor progression and bone disease in a mouse model of multiple myeloma and overcomes resistance to proteasome inhibitors. Blood. (2018) 132:1918. doi: 10.1182/blood-2018-99-117852

[127] Ha KB Lee ES Park NW Jo SH Shim S Kim D-K. Beneficial effects of a curcumin derivative and transforming growth factor-β receptor I inhibitor combination on nonalcoholic steatohepatitis. Diabetes Metab J. (2023) 47:500–13. doi: 10.4093/dmj.2022.0110 PMC10404525 37096379

[128] Kumar V Sethi B Staller DW Xin X Ma J Dong Y. Anti-miR-96 and hh pathway inhibitor MDB5 synergistically ameliorate alcohol-associated liver injury in mice. Biomaterials. (2023) 295:122049. doi: 10.1016/j.biomaterials.2023.122049 36827892 PMC9998370

[129] Kang SH Yim HJ Hwang J Kim M Lee Y-S Jung YK. Improved anti-fibrotic effects by combined treatments of simvastatin and NS-398 in experimental liver fibrosis models. Korean J Intern Med. (2022) 37:745–56. doi: 10.3904/kjim.2021.138 PMC9271712 35811365

[130] Huang S Ding D Lan T He G Ren J Liang R. Multifunctional nanodrug performs sonodynamic therapy and inhibits TGF-β to boost immune response against colorectal cancer and liver metastasis. Acta Biomater. (2023) 164:538–52. doi: 10.1016/j.actbio.2023.04.001 37037269

[131] Luo X Huang W Li S Sun M Hu D Jiang J. SOX12 facilitates hepatocellular carcinoma progression and metastasis through promoting regulatory T-cells infiltration and immunosuppression. Adv Sci. (2024) 11:2310304. doi: 10.1002/advs.202310304 PMC11423149 39072947

[132] Xiang S Li J Zhang M. TGF-β1 inhibitor enhances the therapeutic effect of microwave ablation on hepatocellular carcinoma. Int J Hyperthermia. (2024) 41:2359496. doi: 10.1080/02656736.2024.2359496 38909985

[133] Gobbo F Martelli F Di Virgilio A Demaria E Sarli G Migliaccio AR. The variation in the traits ameliorated by inhibitors of JAK1/2, TGF-β, P-selectin, and CXCR1/CXCR2 in the Gata1low model suggests that myelofibrosis should Be treated by these drugs in combination. Int J Mol Sci. (2024) 25:7703. doi: 10.3390/ijms25147703 39062946 PMC11277099

[134] Liu CH Lee HS Liou JP Hua HS Cheng WH Yuliani FS. MPT0E028, a novel pan-HDAC inhibitor, prevents pulmonary fibrosis through inhibition of TGF-β-induced CTGF expression in human lung fibroblasts: Involvement of MKP-1 activation. Eur J Pharmacol. (2024) 977:176711. doi: 10.1016/j.ejphar.2024.176711 38839029

[135] Byun JK Jung GS. Gemigliptin mitigates TGF-β-induced renal fibrosis through FGF21-mediated inhibition of the TGF-β/Smad3 signaling pathway. Biochem Biophys Res Commun. (2024) 733:150425. doi: 10.1016/j.bbrc.2024.150425 39053104

